# A European multicentre PET study of fibrillar amyloid in Alzheimer’s disease

**DOI:** 10.1007/s00259-012-2237-2

**Published:** 2012-09-08

**Authors:** Agneta Nordberg, Stephen F. Carter, Juha Rinne, Alexander Drzezga, David J. Brooks, Rik Vandenberghe, Daniela Perani, Anton Forsberg, Bengt Långström, Noora Scheinin, Mira Karrasch, Kjell Någren, Timo Grimmer, Isabelle Miederer, Paul Edison, Aren Okello, Koen Van Laere, Natalie Nelissen, Mathieu Vandenbulcke, Valentina Garibotto, Ove Almkvist, Elke Kalbe, Rainer Hinz, Karl Herholz

**Affiliations:** 1 Karolinska Institutet, Stockholm, Sweden; 2 Karolinska University Hospital, Stockholm, Sweden; 3 Turku PET Centre, University of Turku, Turku, Finland; 4 Turku University Hospital, Turku, Finland; 5 Technische Universität München, Munich, Germany; 6 Imperial College London, London, UK; 7 Katolieke Universiteit Leuven, Leuven, Belgium; 8 Vita Salute San Raffaele University, Milan, Italy; 9 University of Uppsala, Uppsala, Sweden; 10 University Clinic of Cologne, Cologne, Germany; 11 University of Vechta, Vechta, Germany; 12 Wolfson Molecular Imaging Centre, University of Manchester, Manchester, UK; 13 Karolinska Institutet, Alzheimer Neurobiology Centre, Geriatric Clinic, Karolinska University Hospital Huddinge, 141 86 Stockholm, Sweden

**Keywords:** Amyloid, Multicentre PET, PIB, MCI, Alzheimer’s disease, Mild cognitive impairment, Cognition

## Abstract

**Purpose:**

Amyloid PET tracers have been developed for in vivo detection of brain fibrillar amyloid deposition in Alzheimer’s disease (AD). To serve as an early biomarker in AD the amyloid PET tracers need to be analysed in multicentre clinical studies.

**Methods:**

In this study 238 [^11^C]Pittsburgh compound-B (PIB) datasets from five different European centres were pooled. Of these 238 datasets, 18 were excluded, leaving [^11^C]PIB datasets from 97 patients with clinically diagnosed AD (mean age 69 ± 8 years), 72 patients with mild cognitive impairment (MCI; mean age 67.5 ± 8 years) and 51 healthy controls (mean age 67.4 ± 6 years) available for analysis. Of the MCI patients, 64 were longitudinally followed for 28 ± 15 months. Most participants (175 out of 220) were also tested for apolipoprotein E (ApoE) genotype.

**Results:**

[^11^C]PIB retention in the neocortical and subcortical brain regions was significantly higher in AD patients than in age-matched controls. Intermediate [^11^C]PIB retention was observed in MCI patients, with a bimodal distribution (64 % MCI PIB-positive and 36 % MCI PIB-negative), which was significantly different the pattern in both the AD patients and controls. Higher [^11^C]PIB retention was observed in MCI ApoE ε4 carriers compared to non-ApoE ε4 carriers (*p* < 0.005). Of the MCI PIB-positive patients, 67 % had converted to AD at follow-up while none of the MCI PIB-negative patients converted.

**Conclusion:**

This study demonstrated the robustness of [^11^C]PIB PET as a marker of neocortical fibrillar amyloid deposition in brain when assessed in a multicentre setting. MCI PIB-positive patients showed more severe memory impairment than MCI PIB-negative patients and progressed to AD at an estimated rate of 25 % per year. None of the MCI PIB-negative patients converted to AD, and thus PIB negativity had a 100 % negative predictive value for progression to AD. This supports the notion that PIB-positive scans in MCI patients are an indicator of prodromal AD.

**Electronic supplementary material:**

The online version of this article (doi:10.1007/s00259-012-2237-2) contains supplementary material, which is available to authorized users.

## Introduction

Alzheimer’s disease (AD) is the most common form of neurodegenerative disorder. Amyloid plaques and neurofibrillary tangles are the pathological hallmarks of AD. It is commonly accepted that AD pathology starts years to decades before the onset of cognitive symptoms [1]. This fact explains why symptomatic AD consistently represents an advanced stage of AD pathology [2]. For future disease-modifying drug therapy there is a need for diagnostic markers allowing detection and diagnosis of AD at the earliest possible stage [3–5].

Recent development of molecular imaging techniques has provided tools to visualize, in vivo, amyloid deposits in the brain with PET. [^18^F]-FDDNP and [^11^C]Pittsburgh compound-B ([^11^C]PIB) were the first amyloid PET tracers developed, and several other PET tracers have then been tested [6, 7]. [^11^C]PIB shows robust retention in AD brain [8, 9] and has been used most widely. High [^11^C]PIB retention has also been observed in patients with mild cognitive impairment (MCI) who later convert to AD [10, 11], and also in elderly cognitively normal individuals [12–14]. Several new ^18^F-labelled amyloid tracers such as [^18^F]florbetaben [15], [^18^F]florbetapir [16], [^18^F]flutemetamol [17] and [^18^F]AZD4694 [18] have been investigated. One of these compounds, [^18^F]florbetapir [19], has already been approved for clinical used in the US by the Food and Drug Administration (FDA). These ^18^F compounds will probably supersede the ^11^C amyloid tracers for routine clinical use in the near future.

The primary aim of this investigation was to determine whether [^11^C]PIB imaging provides consistent findings in a large population of patients with MCI and mild AD examined at different European AD research centres. A secondary aim was to determine the importance of [^11^C]PIB retention to clinical outcome in MCI patients recruited from different centres.

## Materials and methods

### Study population

After data exclusions (see the Image Analysis section below), 97 patients who met the NINCDS-ADRDA criteria for probable AD and the DSM-IV criteria for dementia of AD type [20, 21], 72 patients who met the Petersen criteria for MCI [22] and 51 age-matched healthy controls were recruited from five different European research centres for AD, and were included in this investigation. The participating centres included; Technische Universität München, Munich, Germany (centre A); Katolieke Universiteit Leuven, Leuven, Belgium (centre B); Imperial College London, London, UK (centre C); Karolinska Institutet, Stockholm, Sweden (centre D); and Turku PET Centre, University of Turku, Finland (centre E). The patients had been referred and assessed according to the clinical routines used at the different centres. Age and gender distributions were comparable between all diagnostic groups in the pooled data (Table 1), but differed slightly between centres (Table 1). Details of patient inclusion criteria and technical scanning parameters differed between centres; these can be found in the following publications: [8–11, 23–25]. The participants included in the current pooled dataset had previously been studied and reported in these publications. The controls were mainly recruited from relatives and carers of patients, and not by advertisement.

**Table 1:** Demographics, ApoE and neuropsychological data. The data are presented as means ± SD or *n*

	Controls	*n*	MCI patients	*n*	AD patients	*n*	*p* value		
							Controls vs. MCI	Controls vs. AD	MCI vs. AD
Age (years)	67.4 ± 6.3	51	67.5 ± 8.1	72	69.2 ± 8.4	97	n.s.		
Centre A	–	–	67.1 ± 6.4	11	67.6 ± 8.4	19			
Centre B	69.7 ± 5.9	15	–	–	73.5 ± 7.0	14			
Centre C	64.3 ± 5.1	14	64.9 ± 11	10	62.8 ± 6.9	10			
Centre D	69.0 ± 7.2	6	63.5 ± 8.1	19	67.9 ± 9.1	32			
Centre E	70.1 ± 6.5	16	71.1 ± 6.2	29	73.1 ± 5.9	20			
Male/female	22/29	51	37/35	72	47/50	97	n.s.		
ApoE ε4 carriers	10	31	34	59	48	85			
Centre A	–	–	7	11	10	19			
Centre B	3	15	–	–	9	14			
Centre C	–	–	–	–	–	–			
Centre D	–	–	12	19	9	20			
Centre E	7	16	15	29	20	20			
MMSE score	29.2 ± 1.1	43	27.1 ± 2.0	72	24.0 ± 3.2	97	***	***	***
Centre A	–	–	25.7 ± 2.4	14	22.6 ± 8.4	19			
Centre B	28.9 ± 1.1	7	–	–	24.4 ± 2.7	14			
Centre C	29.8 ± 0.4	14	27.8 ± 1.3	10	24.2 ± 1.5	10			
Centre D	30.0 ± 0	6	28.0 ± 2.1	19	25.6 ± 3.1	34			
Centre E	28.5 ± 1.3	16	26.9 ± 1.5	29	24.1 ± 2.5	20			
Verbal memory immediate (Z)	0.6 ± 1.0	36	−1.0 ± 1.7	64	−1.9 ± 1.4	90	***	***	***
Verbal memory delayed (Z)	0.9 ± 0.8	38	−1.2 ± 1.5	64	−2.2 ± 1.2	90	***	***	***
Nonverbal memory delayed (Z)	0.7 ± 1.2	28	−0.8 ± 1.3	60	−1.7 ± 1.0	71	***	***	***
Visuoconstruction (Z)	0.9 ± 0.6	28	−0.3 ± 1.7	60	−1.0 ± 1.8	70	**	***	n.s.
Verbal fluency (Z)	0.3 ± 1.0	30	−0.7 ± 1.2	44	−1.1 ± 1.0	50	***	***	n.s.
Trail making test A (percentiles)	35.8 ± 32.8	37	19.5 ± 26.7	49	13.4 ± 17.9	74	*	***	n.s.
Trail making test B (percentiles)	44.9 ± 28.4	36	19.1 ± 27.5	49	10.8 ± 21.2	74	***	***	n.s.

*n.s.* not significant, *p < 0.05, **p < 0.01, ***p < 0.001.

*MMSE* minimental state examination, *Z* Z score

### Neuropsychological testing

The Mini-mental state examination (MMSE) score was used as an indicator of general cognitive state. To assess verbal short-term and long-term memory, different tests were used across the centres, including word list learning with immediate and delayed recall with the Rey Auditory Verbal Learning test, and subtests from the ADAS-cog and the CERAD test batteries. Scores for these tests were standardized by Z-transformation according to respective age-matched normative values from the test manuals. Other domains were assessed according to local test protocols. Demographic, diagnostic and neuropsychological data were pseudo-anonymized and transferred to Cologne (Germany) for central evaluation. Not all participants completed all neuropsychological tests; the numbers for each test are displayed in Table 1 (and the centre-by-centre breakdown is displayed in Supplementary Table 1).

### Synthesis of [^11^C]PIB and PET data acquisition

[^11^C]PIB was synthesized using a previously described method [9, 26] at the individual centres according to good manufacturing practice requirements. The following injected doses of [^11^C]PIB and image acquisition parameters were used: *Centre A* Siemens HR+, six 5-min frames 40–70 min after injection of 345 ± 9 MBq; *Centre B* Siemens HR+, 90-min dynamic acquisition after injection of 230 ± 77 MBq; *Centre C* Siemens HR+, 90-min dynamic acquisition after injection of 368 ± 19 MBq; *Centre D* Siemens HR+, 60-min dynamic acquisition after injection of 302 ± 63 MBq; *Centre E* GE Advance, 90-min dynamic acquisition after injection of 458 ± 54 MBq. Supplementary Table 2 shows the PET image reconstruction information for each centre.

### Image analysis

Dynamic [^11^C]PIB PET imaging data were pseudo-anonymized and submitted to the Wolfson Molecular Imaging Centre (Manchester, UK) for central processing. For most participants (151), a T_1_-weighted structural MR image was acquired at 1.5 T, and was also submitted. After exclusion of data from scans with excessive head motion or other artefacts, data from 238 PET and 151 MRI scans remained for processing and analysis, which were performed blinded to clinical diagnosis. [^11^C]PIB data acquired 40–60 min after injection were used, this time window was adopted because it was the maximal acquisition period common to all the participating centres. For each individual participant, [^11^C]PIB frames between 40 and 60 min were summed creating a 40–60-min integral [^11^C]PIB image, which was used for all subsequent processing and analysis.

First, all [^11^C]PIB images corresponding to the 151 available MR images were coregistered and resliced by rigid body transformation (using SPM5; Functional Imaging Laboratory, Wellcome Department of Imaging Neuroscience, UCL, London; [27]) to their respective MR images in native space. Using SPM5, all available MR images (151 in total) were spatially normalized into Montreal Neurological Institute (MNI) space and segmented into grey and white matter tissue classes using the unified segmentation method [28]. As a result of the segmentation, a nonlinear spatial transformation parameter file was created. This parameter file was used to nonlinearly spatially normalize all 151 coregistered [^11^C]PIB integral images from native MRI space to MNI template space.

A subset of ten MR images was used from one centre, centre C, to create a binarized anatomical mask that defined cerebellar and neocortical regions. Data from one centre were used to maintain a level of homogeneity in the MRI data, as each centre had used different MRI scanners and acquisition protocols. Centre C was chosen because it provided the highest quality MRI data (minimal artefacts and excellent grey-to-white matter contrast). The subset of images from centre C contained a mixture of controls, and MCI and AD patients. Summing the ten individual grey matter tissue classes and thresholding at 50 % created a binarized grey matter mask. This thresholded grey matter mask was then further eroded by two voxels in all dimensions to give a closer representation of true grey matter voxels. The resulting eroded grey matter mask was multiplied using a standard digital atlas [29] to create 23 anatomically defined grey matter regions of interest in MNI space.

The accuracy of cerebellar grey matter region placement was checked visually in all participants. In some individuals, cerebellar regions included the lowermost PET slices; these regions show increased voxel variability due to poor count-rate statistics in 3D mode reconstruction. Therefore, median regional [^11^C]PIB cerebellar retention values were obtained. The median voxel value (and not the mean) was chosen as it is insensitive to outliers in low count-rate areas. All the 40–60-min [^11^C]PIB images were then divided by their respective median cerebellar grey matter voxel value to generate [^11^C]PIB retention ratio images. After scaling the individual images, an average image was created resulting in a sample-based PIB template in MNI space based on the 151 coregistered, nonlinearly spatially normalized and scaled [^11^C]PIB PET images.

Eventually, all 238 available 40–60-min [^11^C]PIB PET images, including data from subjects without a structural T_1_-weighted MRI scan, were nonlinearly spatially normalized to the new population-based [^11^C]PIB template using SPM5 with visual control of normalization results.

Following spatial normalization, a further 17 datasets had to be excluded from analysis, 11 because spatial normalization failed and 6 because more than 25 % of the cerebellar reference region in template space was outside the actual field of view of those images. One further subject was excluded because of being diagnosed with frontotemporal dementia at the clinical follow-up examination. Thus, image processing resulted in regional cortical grey matter [^11^C]PIB retention values relative to the cerebellar grey matter of 220 individuals.

### Statistical methods

Variables were analysed using linear regression, ANOVA, and combinations of those in general linear models (GLM) as indicated in the Results section. Differences in distribution of data were analysed with Pearson’s chi-squared test. Kaplan-Meier analysis was used for analysis of dementia-free survival in MCI patients. All procedures were performed using SPSS for Windows (version 16.0).

## Results

### Overall demographic and neuropsychological data

Table 1 shows the demographic data of the participating AD and MCI patients and healthy controls. Overall there was no significant difference in mean age and gender observed among the AD and MCI patients and healthy controls. There was a slight difference between centres in terms of age (*p* = 0.05), but within centres there was no difference in age between the diagnostic groups. No difference in dementia severity in the AD groups was observed between centres and the average MMSE score (24.0 ± 3.2) represented mild AD. The MCI patients recruited from the different centres varied somewhat in MMSE score (mean 26–28). Overall, a higher proportion of subjects with the apolipoprotein E (ApoE) ε4 allele were observed among the AD and MCI patients compared to controls (Supplementary Table 1).

### Comparison of [^11^C]PIB retention among diagnostic groups and centres

**Fig. 1:**
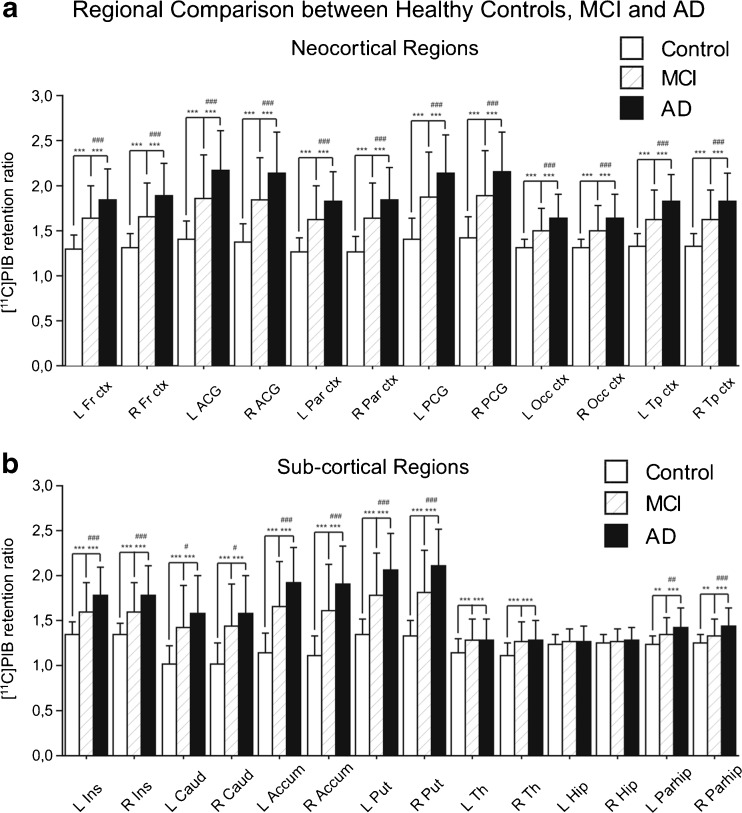
PIB retention ratios in cortical (**a**) and subcortical (**b**) brain regions in 51 healthy controls, 72 patients with MCI and 97 patients with AD. Regional [^11^C]PIB retention is expressed as retentions relative to cerebellar grey matter. Mean values ± SD are shown. *p < 0.05, **p < 0.01, ***p < 0.001, controls versus MCI and controls versus AD; ^#^ *p* < 0.05, ^##^ *p* < 0.01, ^###^ *p* < 0.001, MCI versus AD

**Fig. 2:**
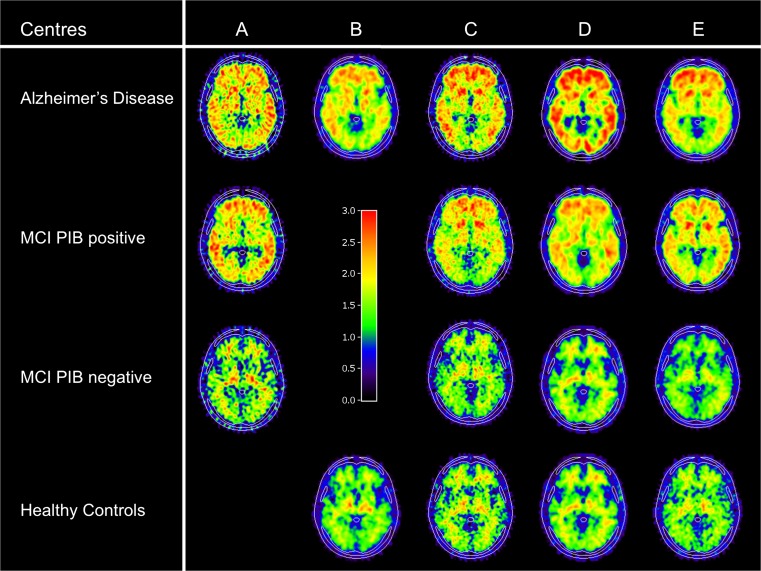
Representative PET scans of healthy controls, patients with MCI and patients with AD from five different centres. The imaging acquisition parameters and mean injected doses used at each centre are presented in the Materials and methods section. Four centres used the HR+ scanner, and centre E used the GE Advance scanner. The images have different visual properties as the centres used different reconstruction parameters, filters and smoothing kernels (see Supplementary Table 2). *Colour scale* PIB retention (*red* high, *green* intermediate, *blue* low)

Figure 1 shows the regional [^11^C]PIB retention (relative to cerebellar grey matter) in the neocortical and subcortical brain regions of the AD and MCI patients and controls. The hippocampus was the only region that did not show a significant difference between the groups. There was a very high correlation of [^11^C]PIB retention across most other brain regions. Thus, we pursued the remainder of the analysis on the average [^11^C]PIB retention in the frontal, parietal and basal/lateral temporal regions, similar to the approaches used by other groups (referred to as neocortical [^11^C]PIB retention) [30]. The [^11^C]PIB retention differed significantly between the three diagnostic groups (AD > MCI > controls; F2,207 = 43.4, *p* < 0.001). Figure 2 shows typical [^11^C]PIB scans of the controls, and AD and MCI patients investigated from the five different PET centres. The variance in [^11^C]PIB retention between centres was eightfold smaller than the variance between diagnostic groups and was not significant in GLM (effect of diagnosis *p* < 0.001, effect of centre and interaction *p* > .0.05).

### Normal controls

**Fig. 3:**
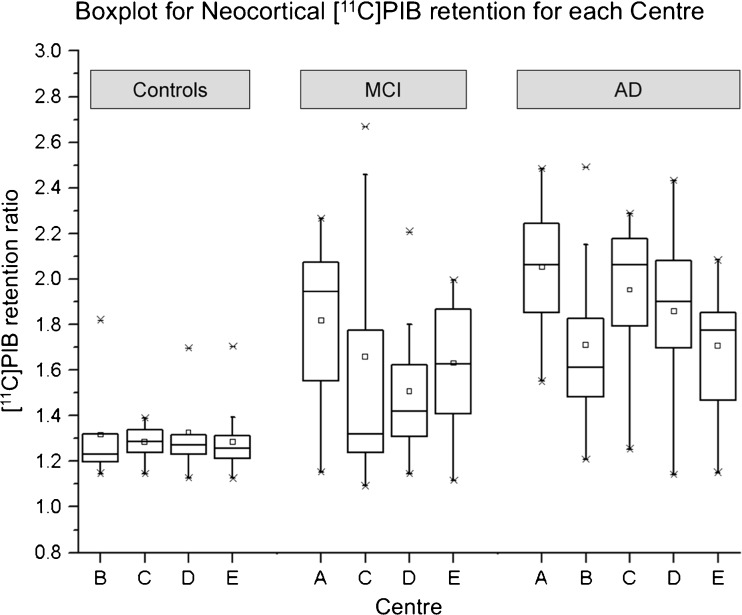
Cortical [^11^C]PIB retention ratios in controls, patients with MCI and patients with AD investigated at five different centres (*A–E*)

**Fig. 4:**
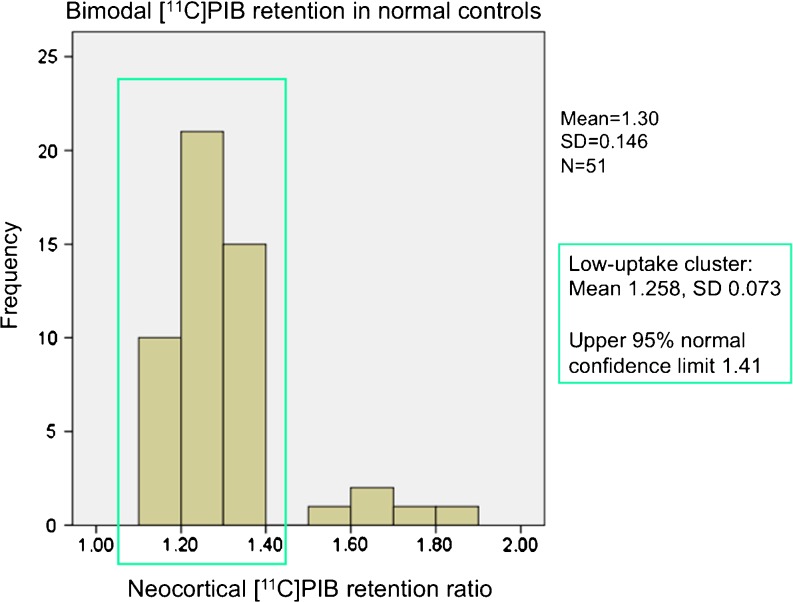
Distribution of neocortical [^11^C]PIB retention ratios in healthy controls. The 95 % upper confidence limit for a normal PIB retention ratio in the healthy controls was defined as 1.41

As demonstrated in Fig. 3 (see also Fig. 4), the vast majority of healthy controls (46 out of 51) showed neocortical [^11^C]PIB retention ratios in the very narrow range of 1.13 to 1.39 (mean 1.26 ± 0.07) and without significant differences between centres. Only five healthy controls from three different centres were clear outliers with regional [^11^C]PIB values above 1.5: three from centre B (1.82, 1.62 and 1.53), one from centre D (1.7) and one from centre E (1.7). The five controls with retention ratios above 1.41 did not differ with respect to demographic characteristics (ages 61 to 77 years; three men, two women; MMSE 26 to 30) from PIB-negative controls. The 46 healthy controls in the main cluster were distributed normally. The upper 95 % confidence limit in the normally distributed control population was 1.41, thus defining the upper normal limit (above which is referred to here as PIB-positive and below which is referred to as PIB-negative).

### AD patients

The mean neocortical [^11^C]PIB retention ratio in AD patients was 1.85 ± 0.32, and 90 % of the AD patients were PIB-positive. PIB-negative patients came from four of the five centres without significant differences in frequency between the centres. Although the level of [^11^C]PIB retention differed somewhat between centres (*p* = 0.003), it was neither related to dementia severity, which was mild and very similar across all centres (MMSE 24 ± 3), nor to ApoE ε4 genotype or patient age.

### MCI patients

The mean neocortical [^11^C]PIB retention ratio in MCI patients was 1.64 ± 0.35, and 65 % of the MCI patients were PIB-positive. There was no significant difference in [^11^C]PIB retention between the centres.

### The effect of apolipoprotein E genotype

Genotyping results were available in 176 of 220 participants (genotyping was unavailable in participants from centre C). As expected, the ApoE ε4 allele was more frequent in the patient groups than in the healthy controls (Table 1). The presence of an ApoE ε4 allele was associated with significantly greater cortical PIB retention (F5,170=7.16, *p*=0.008; interaction with diagnosis *p*=0.018). Within the diagnostic groups, the effect was significant in the MCI group (F2,56=8.1, *p*=0.005), but absent in the AD patients. Among the healthy controls, three of the five PIB-positive subjects carried the ApoE ε4 allele (genotyping was unavailable from one of the PIB-positive controls). There was a significant difference with regard to frequency of the ApoE ε4 allele in AD patients between the centres (*p* = 0.02): centres D and E had a much higher proportion of ApoE ε4 carriers. The ApoE ε4 frequency in the MCI patients showed no significant difference between centres (data from centres A, D and E only) nor in the healthy controls (data from centres B and E only).

### [^11^C]PIB retention and cognitive impairment

Across the entire sample, neocortical [^11^C]PIB retention correlated closely with verbal long delay free recall memory (*r* = −0.60, *p* < 0.001, *n* = 192), as well as with MMSE score (*r* = −0.45, *p* < 0.001). Within the diagnostic groups, long delay free recall was most strongly impaired with increasing [^11^C]PIB retention in the MCI patients (regression slope −2.34, SE 0.54, *p* < 0.001), and less so in the AD patients (slope −0.82, SE 0.38, *p* = 0.04). There was no significant relationship between the MMSE and memory scores and PIB retention in the controls. When the analysis was restricted to PIB-positive patients only, the significant relationship between the MMSE and the long delay free recall and the amount of [^11^C]PIB retention disappeared.

### Longitudinal follow-up in MCI patients

**Fig. 5:**
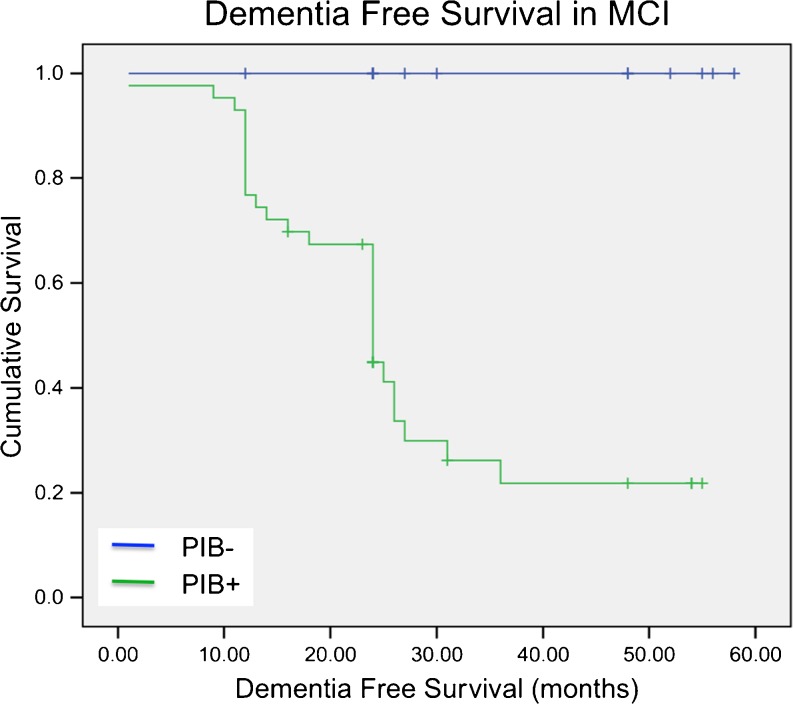
Dementia-free survival in 64 patients with MCI at 28 ± 15 months. None of 21 MCI PIB-negative patients converted to AD while 67.4 % of the MCI PIB-positive patients converted to AD. No difference in conversion rate was observed between MCI patients with relatively high (retention ratio above median) and lower (but retention ratio still >1.41) [^11^C]PIB retention

Clinical follow-up data were available in 64 MCI patients, with a mean follow-up time of 28 ± 15 months. Out of 43 MCI PIB-positive patients, 67.4 % converted (Kaplan-Meier plot, *p* < 0.001, log-rank Mantel-Cox test) to clinical AD while none of the 21 MCI PIB-negative patients (i.e. retention ratio <1.41), converted to AD during follow-up (Fig. 5). Estimated mean dementia-free survival in the MCI PIB-positive group was 27 ± 3 months (median 24 months), corresponding to an annual conversion rate to AD of approximately 25 %. There were no significant differences in dementia-free survival between centres, with estimates of mean survival time by centre ranging between 20 and 31 months. Survival times also did not differ significantly between MCI PIB-positive subjects with relatively high (above median retention ratio >1.85) and relatively low but still above normal (retention ratio >1.41) retention.

## Discussion

The present investigation demonstrated that in a large well-matched population (220 participants) of healthy controls, and MCI and AD patients, consistent complementary PET and clinical data can be acquired from multiple independent Alzheimer centre research sites. When all the data from the five different centres was pooled some heterogeneity was revealed between datasets; namely, in the age, frequency of ApoE ε4 in the AD groups and the cognitive status of the MCI patients. In spite of the differences between centres, the composite neocortical [^11^C]PIB retention data were robust and reliable. Overall across all centres and participant groups the variance explained by the diagnosis was eight times greater than the variance explained by centre alone, with the effect of diagnosis highly significant (*p* < 0.001) and the effect of centre not significant. The variance in patient characteristics between centres was probably an indication of the differences in referral pathways in each centre.

Using the pooled [^11^C]PIB data, the healthy controls showed a bimodal distribution of [^11^C]PIB retention ratios. Our definition of the normal upper limit of neocortical [^11^C]PIB retention ratio was based on identifying the majority subgroup of controls with normally distributed [^11^C]PIB retention in accordance with previous studies [31, 32]. In our sample, only 10 % of the healthy controls were PIB-positive. This proportion is similar to the findings reported by Mintun et al. [33] but lower than the 22 % reported by Pike et al. [32] and the 33 % reported by Rowe et al. [14]; these studies used slightly higher thresholds for [^11^C]PIB positivity (1.6 and 1.5 respectively). The study by Rowe et al. [14] used partial volume correction (which normally increases the mean regional values of PET data) and also had a significantly older healthy control population similar to the other studies with a higher proportion of PIB-positive controls [31, 34–36]. It has been clearly established in multiple studies that there is a strong relationship between age and [^11^C]PIB retention, so it is unsurprising that the younger healthy control population investigated here showed lower [^11^C]PIB retention ratios. The frequency of 65 % PIB-positive in MCI and 90 % in AD are comparable to the values found in the aforementioned studies.

Overall, the MCI patients formed a cognitively heterogeneous population with the majority probably having amnestic multidomain MCI [37]. Likewise, the MCI patients had heterogeneous neocortical [^11^C]PIB retention and could be split into PIB-positive and PIB-negative subgroups. None of the latter group (21 patients) converted to AD during follow-up, and thus PIB negativity had a 100 % negative predictive value for progression to AD. The cognitive deficit in PIB-negative patients with MCI may have been due to causes unrelated to amyloid pathology. Two-thirds (67.4 %) of the MCI PIB-positive patients converted (29 patients) during the longitudinal follow-up and no significant difference in progression were observed between the PIB-positive patients who had very high [^11^C]PIB retention (retention ratio >1.85) and moderate retention (retention ratio >1.41, <1.85). The estimated median dementia-free survival time in the MCI PIB-positive patients was 27 months, corresponding to an annual conversion rate of approximately 25 %, while the annual conversion rate to AD is typically in the 10–15 % range in unselected MCI populations from a memory clinic.

In two earlier studies [10, 11] of patient subgroups that were included in this study, up to 82 % of MCI PIB-positive patients converted to AD within 3 years. These two studies did not estimate annual conversion rates. It is well documented that conversion rates in MCI are related to MCI subtype, severity of the memory deficit and ApoE genotype [37]. It is therefore very difficult to directly compare different MCI studies, and reported progression rates vary considerably. Our investigation represents pooled data based on referrals from various specialized memory clinics across Europe and is therefore likely to be informative for clinical trial samples drawn from similar institutions.

The MCI group showed the strongest correlation between [^11^C]PIB retention and memory, while this correlation was weakest in healthy controls. Restricting the correlation to PIB-positive patients only, the significant correlation with cognitive state and memory disappeared, suggesting that neuropsychological deficits in these patients are associated with the presence of fibrillar amyloid deposits in a binary manner and do not depend on the quantitative amount. This is in agreement with the findings of other studies that have demonstrated a limited relationship between amyloid load and cognition [31, 38, 39].

Relatively low and nonsignificant hippocampal [^11^C]PIB retention was found in the three groups (Fig. 1). It is known that fibrillar amyloid pathology in the hippocampus is limited relative to the neocortex and that the hippocampus is more susceptible to neurofibrillary tangle pathology [2]. There are recent reports that suggest [^11^C]PIB retention is increased in the hippocampus in MCI and AD and it reflects the region’s susceptibility to amyloid toxicity and subsequent cognitive deficits [40, 41]. A likely explanation for the difference in the current investigation is that it used a PET-based template compared to the aforementioned studies that used structural MRI data to process and analyse the [^11^C]PIB data. It is possible in the current investigation that sampling regions of lower amyloid in the medial temporal lobe (specifically the hippocampus) led to underestimation of [^11^C]PIB retention, particularly in MCI and AD patients who have hippocampal atrophy. Without the support of a structural MRI image to aid spatial normalization in these regions of low [^11^C]PIB retention, the partial volume effect in the hippocampus is likely to be exacerbated. This suggestion certainly warrants further testing in the 151 datasets used in the current investigation that have a structural MRI scan available; however, it is beyond the scope of the current article.

As has been reported previously [14, 25, 32, 42], the presence of at least one ApoE ε4 allele was associated with increased neocortical [^11^C]PIB retention. In the current sample the effect was significant in the MCI patients, but not the AD patients or the healthy controls. Only 32 % of the healthy controls carried at least one ApoE ε4 allele, and no healthy controls were ApoE ε4 homozygous. This finding also provides a further explanation, in addition to age, as to why there were fewer PIB-positive healthy controls in the current population than in the study by Rowe et al. [14]. For example, in that study 43 % of subjects carried at least one ApoE e4 allele of whom many individuals were ApoE ε4 homozygous. In the current investigation, three of the five healthy controls who were PIB-positive carried one ApoE ε4 allele, but at the time of investigation showed preserved cognition. It is, however, possible that these individuals may go on to experience cognitive impairment and later develop AD. Longitudinal investigation would be necessary to determine the validity of this hypothesis.

From a methodological perspective, the analysis of the [^11^C]PIB data was restricted to static retention ratios, which are more practical for multicentre studies than parameters derived from full kinetic analysis. Static retention values provide a robust and sensitive parameter [43] that is highly correlated with other kinetically derived parameters [44]. The standardized automated procedure that was developed robustly determined regional [^11^C]PIB retention, demonstrating its feasibility for efficient image processing in multicentre studies. There was, however, a small but significant difference between centres in the group [^11^C]PIB retention values. This was probably to have been due to the different PET scanners and image reconstruction algorithms used in the participating centres in combination with the slight differences in demographics observed between centres. Harmonized referral pathways, image acquisition and reconstruction protocols for centrally funded prospective studies and clinical trials would reduce such methodological differences.

### Conclusion

This investigation into amyloid PET using [^11^C]PIB included a large sample taken from multiple AD centres using different scanners, different referral pathways and different implementations of standard inclusion criteria for MCI. The normal range of [^11^C]PIB retention was narrow and robust across centres, and increased retention was present in the vast majority of AD patients. MCI patients showed intermediate retention on average with more than half of the subjects showing AD-type patterns. A PIB-positive PET scan can identify MCI patients with a high risk of converting to AD, and a PIB-negative finding had a very high negative predictive value excluding progression to AD. It is highly likely that ^18^F amyloid compounds will replace ^11^C compounds such as [^11^C]PIB in general clinical practice following the FDA’s approval in the US of [^18^F]florbetapir for clinical use [19]. However, the knowledge obtained from the current investigation will be of great importance, particularly in view of its multicentre setting. The variance of [^11^C]PIB retention between different participating centres was low compared to the large differences between diagnostic groups, suggesting that results obtained from [^11^C]PIB PET are highly consistent and reproducible. A similar paradigm should certainly be applied to the new ^18^F compounds as data become available from multiple research sites. What this investigation clearly demonstrates is that amyloid imaging is both a highly useful tool for diagnosis of AD in its earliest symptomatic stages and is suitable for identifying patients for antiamyloid therapy in multicentre clinical trials.

## Electronic supplementary material

Below is the link to the electronic supplementary material.

Supplementary Table 1 MMSE, ApoE genotype and neuropsychological test performance from each centre (DOC 69 kb)

Supplementary Table 2 (DOC 38 kb)
