# Supplementary material for: A European multicentre PET study of fibrillar amyloid in Alzheimer’s disease
Source: Eur J Nucl Med Mol Imaging. 2012 Sep 8;40(1):104–14. doi: 10.1007/s00259-012-2237-2 (PMC3510420; doi:10.1007/s00259-012-2237-2)
Supplement: Supplementary file 1 — MMSE, ApoE genotype and neuropsychological test performance from each centre (DOC 69 kb) [file 259_2012_2237_MOESM1_ESM.doc]

Supplementary Table 1: MMSE, ApoE genotype and neuropsychological test performance from each centre

|  |  | **Group** | **Centre A** | **Centre B** | **Centre C** | **Centre D** | **Centre E** | **Centres total** |
| --- | --- | --- | --- | --- | --- | --- | --- | --- |
| **General cognitive state** | **MMSE (raw scores)** | **Control** | [n=0] | 28.9 (1.1) [n=7] | 29.8 (0.4) [n=14] | 30.0 (0.0) [n=6] | 28.5 (1.3) [n=16] | 29.2 (1.1) [n=43] |
|  |  | **MCI** | 25.7 (2.4) [n=14] | [n=0] | 27.8 (1.3) [n=10] | 27.9 (2.1) [n=19] | 26.9 (1.5) [n=29] | 27.1 (2.0) [n=72] |
|  |  | **AD** | 22.6 (4.4) [n=19] | 24.4 (2.7) [n=14] | 24.2 (1.5) [n=10] | 24.6 (3.1) [n=34] | 24.2 (2.5) [n=20] | 24.0 (3.2) [n=97] |
| **ApoE ε4 carriers** | **(homo- & heterozygous)** | **Control** | [n=0] | 3 [n=15] | [n=0] | [n=0] | 7 [n=16] | 10 [n=31] |
|  |  | **MCI** | 7 [n=11] | [n=0] | [n=0] | 12 [n=19] | 15 [n=29] | 34 [n=59] |
|  |  | **AD** | 10 [n=19] | 9 [n=14] | [n=0] | 9 [n=32] | 20 [n=20] | 48 [n=85] |
| **Verbal Memory** | **AVLT or ADAS or CERAD subtest (Z-scores)** | **Test** | **CERAD** | **AVLT** | **ADAS** | **AVLT** | **CERAD** |  |
|  |  | **Control** | [n=0] | 0.9 (0.9) [n=15] | 0.4 (0.8) [n=7] | [n=0] | 1.02 (0.8) [n=16] | 0.9 (0.8) [n=38] |
|  |  | **MCI** | -2.1 (1.3) [n=14] | [n=0] | -0.2 (1.9) [n=2] | -0.9 (1.1) [n=19] | -0.9 (1.8) [n=29] | -1.2 (1.5) [n=64] |
|  |  | **AD** | -2.6 (1.1) [n=16] | -1.9 (0.7) [n=14] | -3.4 (0.9) [n=9] | -2.0 (0.9) [n=31] | -1.8 (1.5) [n=20] | -2.2 (1.2) [n=90] |
| **Speed of information processing/**  **Executive functions** | **TMT A (percentiles)** | **Control** | [n=0] | 48.1 (28.6) [n=15] | 70.8 (17.2) [n=6] | [n=0] | 11 (20.1) [n=16] | 35.8 (32.8) [n=37] |
|  |  | **MCI** | [n=0] | [n=0] | 50.0 (45.2) [n=2] | 39.7 (29.3) [n=18] | 4.9 (8.9) [n=30] | 19.5 (26.7) [n=49] |
|  |  | **AD** | [n=0] | 22.5 (20.6) [n=14] | 10.6 (16.1) [n=9] | 15.2 (19.7) [n=31] | 5.6 (8.8) [n=23] | 13.4 (17.9) [n=74] |
|  | **TMT B (percentiles)** | **Control** | [n=0] | 48.4 (28.8) [n=15] | 81.6 (18.0) [n=5] | [n=0] | 30.1 (18.2) [n=16] | 44.9 (28.4) [n=36] |
|  |  | **MCI** | [n=0] | [n=0] | 63.0 (31.1) [n=2] | 34.3 (31.9) [n=18] | 6.6 (14.0) [n=29] | 19.1 (27.5) [n=49] |
|  |  | **AD** | [n=0] | 9.1 (16.3) [n=14] | 8.9 (26.7) [n=9] | 11.8 (24.7) [n=31] | 11.2 (16.7) [n=20] | 10.8 (21.2) [n=74] |
|  | **Verbal fluency: “Animals” (Z-scores)** | **Test** | **Animals** | **Animals** | **Animals** |  | **Animals** |  |
|  |  | **Control** | [n=0] | 0.05 (1.0) [n=7] | -0.2 (1.1) [n=7] | [n=0] | 0.6 (0.9) [n=16] | 0.3 (1.0) [n=30] |
|  |  | **MCI** | -1.1 (0.9) [n=13] | [n=0] | -0.8 (0.7) [n=2] | [n=0] | -0.5 (1.3) [n=29] | -0.7 (1.2) [n=44] |
|  |  | **AD** | -1.4 (1.0) [n=16] | -1.2 (0.4) [n=5] | -1.7 (0.6) [n=9] | [n=0] | -0.5 (1.0) [n=20] | -1.1 (1.0) [n=50] |
| **Visuocon-struction** | **RCF, or CERAD subtest (Z-scores)** | **Test** | **RCFT** | **RCFT** | **RCFT (other version)** | **RCFT** | **CERAD subtest** |  |
|  |  | **Control** | [n=0] | 1.4 (0.3) [n=12] | [n=0] | [n=0] | 0.5 (0.5) [n=16] | 0.9 (0.6) [n=28] |
|  |  | **MCI** | -0.9 (1.6) [n=13] | [n=0] | [n=0] | -0.7 (2.1) [n=18] | 0.1 (1.2) [n=29] | -0.3 (1.7) [n=60] |
|  |  | **AD** | -0.8 (1.8) [n=16] | 0.6 (1.6) [n=5] | [n=0] | -1.9 (2.0) [n=29] | -0.3 (1.8) [n=20] | -1.0 (1.8) [n=70] |
| **Non-Verbal memory** | **RCF, delayed recall, or CERAD subtest (Z-scores)** | **Test** | **RCFT** | **RCFT** | **RCFT (other version)** | **RCFT** | **CERAD** |  |
|  |  | **Control** | [n=0] | 1.4 (1.1) [n=12] | [n=0] | [n=0] | 0.2 (0.9) [n=16] | 0.7 (1.2) [n=28] |
|  |  | **MCI** | -1.8 (0.9) [n=12] | [n=0] | [n=0] | -0.8 (1.5) [n=19] | -0.4 (1.0) [n=29] | -0.8 (1.3) [n=60] |
|  |  | **AD** | -2.2 (0.8) [n=15] | -1.1 (0.9) [n=5] | [n=0] | -2.0 (0.9) [n=31] | -0.9 (0.9) [n=20] | -1.7 (1.0) [n=71] |

AVLT: Auditory Verbal Learning Test; ADAS: Alzheimer´s Disease Assessment Scale; TMT: Trail Making Test; RCFT: Rey Complex Figure Test
