# Supplementary material for: A European multicentre PET study of fibrillar amyloid in Alzheimer’s disease
Source: Eur J Nucl Med Mol Imaging. 2012 Sep 8;40(1):104–14. doi: 10.1007/s00259-012-2237-2 (PMC3510420; doi:10.1007/s00259-012-2237-2)
Supplement: Supplementary file 2 — (DOC 38 kb) [file 259_2012_2237_MOESM2_ESM.doc]

**Supplementary Table 2**

| **Centre** | **Scanner** | **Image matrix** | **Voxel size (mm)** | **Reconstruction** |
| --- | --- | --- | --- | --- |
| **Centre A** | Siemens HR+ | 128x128x63 | 2.06x2.06x2.425 | 3D scanning mode; attenuation corrected; FBP, cut-off frequency 0.3; Ramp 03 filter |
| **Centre B** | Siemens HR+ | 128x128x63 | 2.06x2.06x2.425 | 3D scanning mode; attenuation, scatter and random corrected; FBP, Hanning filter (6mm) |
| **Centre C** | Siemens HR+ | 128x128x63 | 2.092x2.092x2.42 | 3D scanning mode; attenuation and scatter corrected; FBP; All pass filter (Ramp, 2mm) |
| **Centre D** | Siemens HR+ | 128x128x63 | 2.06x2.06x2.425 | 3D scanning mode; attenuation, scatter and dead-time corrected; FBP; Hanning filter (4mm) |
| **Centre E** | GE Advance | 128x128x35 | 2.34x2.34x4.25 | 3D scanning mode; attenuation corrected; FBP; transaxial Hann filter (4.6mm); axial Ramp filter (8.5mm) |

FBP = filtered back projection
